# Inhibiting Glycolysis and ATP Production Attenuates IL-33-Mediated Mast Cell Function and Peritonitis

**DOI:** 10.3389/fimmu.2018.03026

**Published:** 2018-12-18

**Authors:** Heather L. Caslin, Marcela T. Taruselli, Tamara Haque, Neha Pondicherry, Elizabeth A. Baldwin, Brian O. Barnstein, John J. Ryan

**Affiliations:** ^1^VCU Life Sciences, Virginia Commonwealth University, Richmond, VA, United States; ^2^Department of Biology, Virginia Commonwealth University, Richmond, VA, United States

**Keywords:** IL-33, mast cells, glycolysis, ATP, metabolism, metformin

## Abstract

Cellular metabolism and energy sensing pathways are closely linked to inflammation, but there is little understanding of how these pathways affect mast cell function. Mast cells are major effectors of allergy and asthma, and can be activated by the alarmin IL-33, which is linked to allergic disease. Therefore, we investigated the metabolic requirements for IL-33-induced mast cell function, to identify targets for controlling inflammation. We found that IL-33 increases glycolysis, glycolytic protein expression, and oxidative phosphorylation (OX PHOS). Inhibiting OX PHOS had little effect on cytokine production, but antagonizing glycolysis with 2-deoxyglucose or oxamate suppressed inflammatory cytokine production *in vitro* and *in vivo*. ATP reversed this suppression. Glycolytic blockade suppressed IL-33 signaling, including ERK phosphorylation, NFκB transcription, and ROS production *in vitro*, and suppressed IL-33-induced neutrophil recruitment *in vivo*. To test a clinically relevant way to modulate these pathways, we examined the effects of the FDA-approved drug metformin on IL-33 activation. Metformin activates AMPK, which suppresses glycolysis in immune cells. We found that metformin suppressed cytokine production *in vitro* and *in vivo*, effects that were reversed by ATP, mimicking the actions of the glycolytic inhibitors we tested. These data suggest that glycolytic ATP production is important for IL-33-induced mast cell activation, and that targeting this pathway may be useful in allergic disease.

## Introduction

Cellular metabolism and energy sensing pathways control the breakdown of carbohydrates, fatty acids, and proteins when energy is required. While we know that cells require energy for homeostasis, maintenance, and proliferation, it is now understood that metabolism is closely linked to immune cell differentiation and activation, impacting cell phenotype, effector functions, and overall inflammatory conditions (1). Glycolysis is consistently noted as the primary energy production pathway used by inflammatory cells, such as T-helper (Th)1, Th17, M1 macrophages, and dendritic cells (DCs) during acute activation, while oxidative phosphorylation (OX PHOS) in the electron transport chain (ETC) is the primary energy production pathway used by regulatory cells such as T-regulatory (Treg), M2 macrophages, and myeloid derived suppressor cells (MDSC) (2–5). Glycolysis is inefficient, with only 2 ATP produced per glucose molecule compared with 32 per glucose in OX PHOS, but the benefits of utilizing glycolysis during activation and proliferation are multi-fold. Glycolysis rapidly increases ATP availability, operates under low oxygen tension, and provides pentose phosphate pathway and Kreb's cycle intermediates to anabolic pathways producing nucleotides, amino acids, and lipids (2, 6). This has been most extensively studied in T cells, which undergo dynamic and complex metabolic reprogramming in response to activation, cytokine stimulation, and other changes in their microenvironment (4, 5, 7). In contrast, there is limited information on how metabolism is modulated in mast cells.

Mast cells are tissue resident myeloid cells that reside in both mucosal and connective tissue. These cells are typically recognized for their effector function in Th2 immunity, specifically their detrimental role in allergic disease and protective role against parasites and venoms (8–11). While much is known about mast cell activation, little data concerning the role of glucose metabolism in mast cell responses has been published. Studies from the 1990's suggest that adequate glucose and ATP are required for full mast cell function (12–14), and that lactate is released upon activation by compound 48/80 and polymyxin B in rat mast cells (15). Additionally, studies by Chakravarty showed that glycolytic blockade with 2-deoxyglucose (2-DG), iodoacetate, fluoride and oxamate (OX) suppressed compound 48/80 and antigen-induced histamine release in rat mast cells (16, 17). Recently, an extracellular flux analyzer (Seahorse device) was used to show that IgE XL rapidly increases glycolysis, while OX PHOS increases ~2 h after stimulation (18). The same study showed that suppressing glycolysis with dichloroacetate (DCA) and inhibiting complex I of the electron transport chain (ETC) with rotenone suppressed cytokine production and degranulation. By contrast, inhibiting fatty acid oxidation with etomoxir had no effect (18). Additionally, OX PHOS activity has been shown to increase following IgE XL in mast cells via p-ERK and mitochondrial Stat3 (19). These results suggest that IgE-mediated activation requires glycolysis and ETC activity, however the metabolic requirements for other important mast cell activators have not been examined.

IL-33 is a cytokine mediator that is considered an alarmin. It is released by endothelial, epithelial, and fibroblast cells in response to damage, and by mast cells following activation (20–22). IL-33 activates many immune cell types, including mast cells, Th2, and innate-like lymphoid cell- (ILC)2. It augments IgE-induced inflammation (23, 24) and is elevated in asthma and atopic dermatitis (25–28). IL-33 administration promotes disease in animal studies, while anti-IL-33 or anti-ST2 antibody treatments can reduce inflammation (29, 30). Thus, understanding its function may be critical to allergic disease. We have recently shown that lactic acid, a byproduct of glycolysis, can suppress IL-33-induced mast cell activation (31). This prompted interest in how metabolism may contribute to IL-33 function, which has not been studied in immune cells.

Our purpose was to determine the metabolic requirements for IL-33 activation in mast cells and to examine potential targets for controlling IL-33-mediated inflammation. Our data suggest that IL-33-induced cytokine secretion requires glycolysis for ATP production and that glycolytic blockade suppresses inflammatory cytokine production *in vitro* and *in vivo*. To test proof of principle and suggest a clinically relevant way to modulate these pathways in humans, we report the effects of the FDA-approved drug metformin on IL-33 activation. AMPK induction by metformin, which suppresses glycolysis in immune cells, inhibited cytokine production *in vitro* and *in vivo*. These data suggest that suppressing glycolysis directly or via AMPK activation has therapeutic potential for IL-33-mediated inflammation.

## Methods

### Animals

Mouse C57BL/6J and NFκB-luc breeding pairs were purchased from The Jackson Laboratory (Bar Harbor, ME), and colonies were maintained in a pathogen free facility. Bone marrow was extracted from mice at a minimum of 10 weeks old and IL-33-induced peritonitis studies were conducted at 10–16 weeks of age with both male and female mice with approval from the Virginia Commonwealth University Institutional Animal Care and Use Committee.

### Mast Cell Culture

Mouse bone marrow cells were differentiated in complete RPMI 1640 media supplemented with WEHI-3 cell supernatant containing IL-3 and BHK-MKL cell supernatant containing SCF as described to yield 90-99% FcεRI^+^ and cKit^+^ bone marrow derived mast cells (BMMC) at 21–28 days (31, 32). Following differentiation and expansion, BMMC were plated at 1x10^6^/mL with IL-3 and SCF for all experiments (10 ng/mL). Cells were treated as described and activated ± IL-33 at 50 ng/mL unless otherwise stated.

### Materials

Recombinant mouse IL-3, SCF, and IL-33 for *in vitro* experiments were purchased from Shenandoah Biotechnology (Warwick, PA). Sodium oxamate and 2-deoxyglucose (2DG) were purchased from Alfa Aesar (Tewksbury, MA). Etomoxir, rotenone, and SRT1720 were purchased from Cayman Chemical (Ann Arbor, MI). Antimycin A was purchased from Chem Cruz via Santa Cruz Biotechnology (Dallas, TX). ATP disodium salt was purchased from Tocris via Biotechne Corporation (Minneapolis, MN). Metformin was purchased from MP Biosciences (Santa Ana, CA). A769662 was purchased from Med Chem Express (Monmouth Junction, NJ).

### *In vivo* Studies

Recombinant mouse IL-33 for *in vivo* experiments was purchased from Biolegend (San Diego, CA). Age- and sex- matched groups of mice (~12 weeks old) were injected intraperitoneally (IP) with 2-DG (1 g/kg, ~100 μl), sodium oxamate (15 mg/kg, ~100 μl), metformin (100 mg/kg, ~100 μl), or PBS (100 μl) 1 h prior to IL-33. IL-33 (1 μg/mouse in 100 μl PBS) was injected IP to elicit peritonitis, and mice were sacrificed after 4 h. Plasma from cardiac puncture was used to measure cytokines via ELISA, and neutrophil recruitment was assessed from peritoneal lavage cells analyzed with flow cytometry as described below.

### Cellular Metabolism

To measure the extracellular acidification rate (ECAR), proton production rate (PPR), and oxygen consumption rate (OCR) as surrogates for glycolysis and oxidative phosphorylation, a Seahorse XFp analyzer (Agilent, Santa Clara, CA) was used. Cells were plated in duplicate at 200,000/well on 4.6 μg/ml Cell-Tak^TM^ in minimal DMEM containing 10 mM glucose, 1 mM sodium pyruvate, 2 mM L-glutamine, and 1% FBS. The protocol was as follows: initialization, 3 cycles baseline, inject IL-3/SCF (10 ng/ml final concentration), 3 cycles, inject IL-33 (100 ng/ml final concentration), 5 cycles. For each condition, an average was taken across all wells.

To determine glucose uptake and lactate export, cell supernatants were analyzed for glucose and lactate concentrations 16 h after activation, using the Glucose Assay Kit 1 and L-Lactate Assay Kit 1 from Eton Bioscience (San Diego, CA). Glucose uptake was calculated as [glucose in unactivated cell supernatant] – [glucose in activated cell supernatant]. Lactate export was calculated as [lactate in activated cell supernatant] – [lactate in unactivated cell supernatant].

### Gel Electrophoresis and Western Blot

To determine protein concentration and protein phosphorylation, cell lysates were collected using Protease arrest (GBiosciences, Maryland Heights, MO) in cell lysis buffer (Cell Signaling Technology, Danvers, MA). Protein concentration was determined using the Pierce BCA Protein Assay Kit (Thermofisher, Waltham, MA). 4–20% Mini-PROTEAN® TGX™ Precast Protein Gels (Bio–Rad, Hercules, CA) were loaded with 30 μg protein, electrophoresed and transferred to nitrocellulose (Pall Corporation, Ann Arbor, MI), and membranes were blocked for 60 min in Blocker casein in tris-buffered saline (TBS) (from Thermofisher, Waltham, MA). Blots were incubated with primary antibodies overnight in block buffer + Tween20 (1:1,000) ± rabbit anti-p-AMPK (1:750), rabbit anti-HK2 (1:750), rabbit anti-actin (1:1,000, antibodies all purchased from Cell Signaling, Danvers, MA). Blots were washed six times for 5 min each in TBS-Tween-20, followed by incubation with secondary antibody (1:10,000) for 60 min at room temperature (Cell Signaling, Danvers, MA). Size estimates for proteins were obtained using molecular weight standards from Bio–Rad (Hercules, CA). Blots were visualized and quantified using a LiCor Odyssey CLx Infrared imaging system (Lincoln, NE). After background subtraction, fluorescence intensity for the protein of interest was normalized to the signal intensity for the relevant loading control and unactivated samples, using Image Studio 4.0 (LiCor).

### ELISA

ELISA analysis was used to measure cytokine concentrations from the cell culture supernatant 16 h after activation and from the plasma 4 h after IL-33 induced peritonitis (described above). Murine IL-6, TNF, and MCP-1 (CCL2) ELISA kits were purchased from Biolegend; murine MIP-1α (CCL3) ELISA kits were purchased from Peprotech (Rocky Hill, NJ). Assays were performed in duplicate (plasma) or triplicate (cell supernatant) according to the manufacturers' protocols.

### Flow Cytometry

For cell signaling studies, cells were activated for 15 min. Cells were collected with 1.6% paraformaldehyde fixation and permeablized with methanol for p-ERK analysis. Cells were stained with anti-CD16/32 (clone 2.4G2, BD Pharmingen via BD Biosciences, San Jose, CA) and APC-anti-H/M pERK1/2 (clone MILAN8R, eBioscience, via Thermofischer, Waltham, MA) or the isotype control (APC mouse IgG1; eBioscience) at 2 μg/mL for 30 min at 4°C, and analyzed by flow cytometry with the FACsCelesta (BD Biosciences). The gating strategy used doublet exclusion (FSC-A x FSC-H), and size vs. granularity (FSC x SSC). MFI was recorded for all samples.

For oxidative stress measures, cells were treated ± 2DG or OX for 1 h then activated with IL-33 for 2 h. Cells were then washed and re-suspended in Hank's buffered saline solution (HBSS) + 2′,7′ Diochlorofluorescin Diacetate (DCFH-DA, 5 μM, Millipore, Burlington, MA) ± 2DG or OX ± IL-33 for 30 min at 37°C. Cells were analyzed in the FITC channel by flow cytometry. The gating strategy used was doublet exclusion (FSC-A x FSC-H) and gating on size and granularity (FSC x SSC). MFI was recorded for all samples.

For ATP diffusion, cells were treated with AlexaFluor 647-labeled ATP (ThermoFisher, Waltham, MA) at 1, 2.5, 5, and 8 μM for 20 min in cRPMI at 37°C. Cells were then washed 2X and suspended in PBS for flow cytometric analysis. Percent positive cells were recorded for all samples.

Following IL-33-induced peritonitis (described above), peritoneal lavage cells were collected, red blood cells were lysed with ACK buffer, and rinsed pellets were stained with anti-CD16/32 (clone 2.4G2, BD Pharmingen), PE rat anti- mouse Ly6G (clone1A8, BD Pharmingen), APC-anti mouse CD45 (clone 30-F11, Biolegend) or the isotype controls PE rat IgG2a (BD Pharmingen) and APC rat IgG2b (Biolegend), all at 2 μg/mL for 30 min at 4°C, and analyzed by flow cytometry with the FACsCelesta (BD Biosciences). The gating strategy used was doublet exclusion (FSC-A x FSC-H), size and granularity (FSC x SSC), lymphocytes (CD45+), and neutrophils (Ly6G++). Percent positive was reported from total leukocyte (CD45+) events.

### Luciferase

BMMC were differentiated from NFκB-luc transgenic bone marrow as above. Following treatment ± 2DG or OX for 1 h and IL-33 activation for 2 h, cells were lysed and luciferase activity was measured with the Promega Luciferase Assay Substrate and Glomax 20/20 Luminometer (Promega, Madison, WI). Luciferase expression is reported relative to protein concentration (Pierce BCA Protein Assay Kit, Thermofisher, Waltham, MA) and normalized to the unactivated control.

### Statistical Analyses

**Figure 1:**
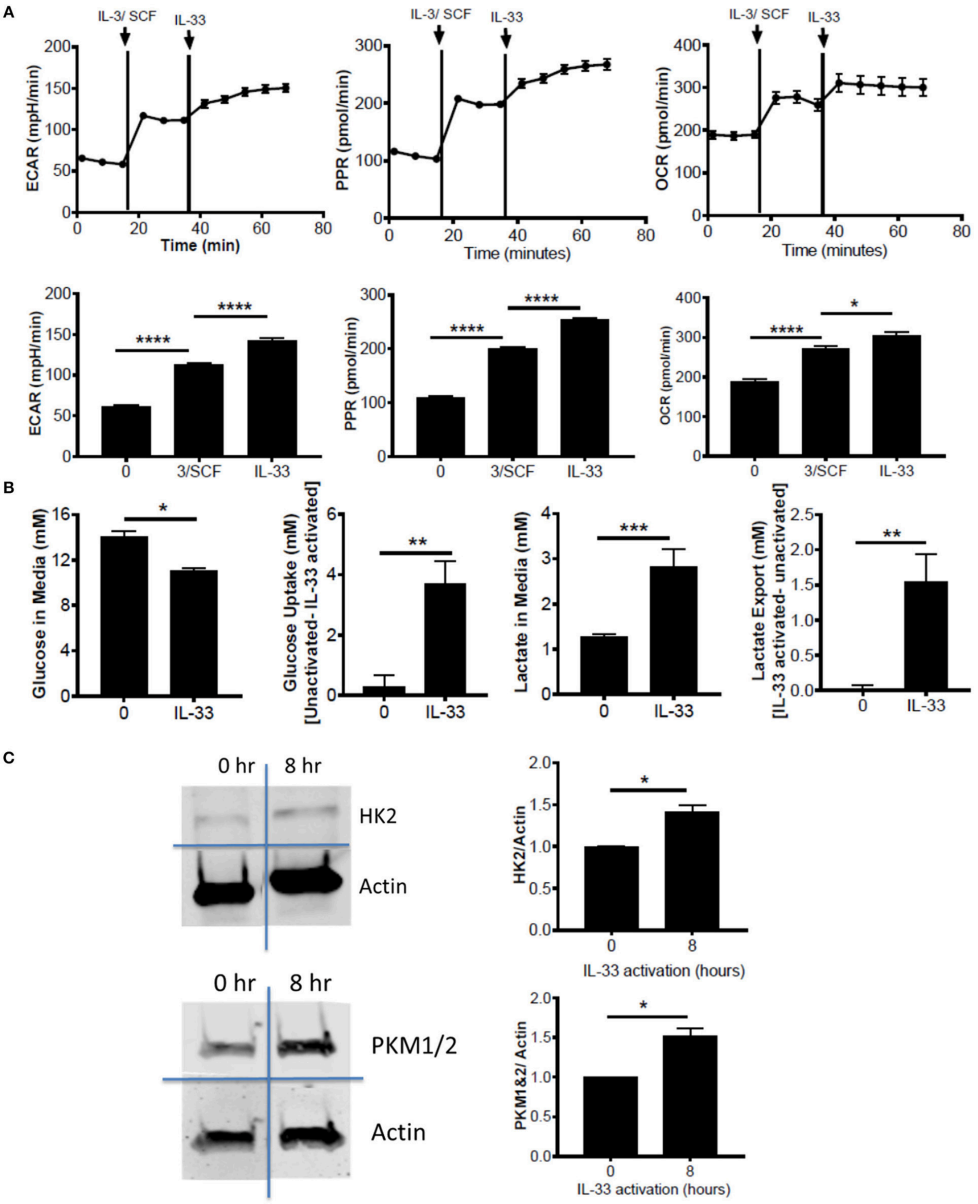
IL-33 activation induces glycolysis and OX PHOS. **(A)** Seahorse metabolic analysis was used to measure basal metabolism in BMMC, followed by IL-3/SCF (10 ng/mL) and IL-33 (100 ng/mL). ECAR, PPR, and OCR measurements averaged over time are shown. **(B)** BMMC were activated for 16 h ± IL-33 (50 ng/ml). Glucose and lactate were measured in the cell supernatant by colorimetric assay. Uptake and export were determined by the difference between activated and unactivated groups. Above data are means ± SEM of 3 populations, representative of 3 independent experiments. **(C)** BMMC were activated with IL-33 (100 ng/ml) for 8 h. Lysates were analyzed by western blot for hexokinase, pyruvate kinase, and actin. Data are representative of 3 independent populations ^*^p < 0.05, ^**^p < 0.01, ^***^p < 0.001, ^****^p < 0.0001.

Glucose uptake, lactate export, and enzyme expression (Figures 1B,C; comparison of two groups) were analyzed by *t*-test. The remainder of the data (3+ groups) were analyzed by a one-way analysis of variance (ANOVA) to detect overall differences between groups. With F-statistic significance, Tukey's multiple comparisons were used as *post hoc* tests to determine which conditions were significantly different from the control. We have reported only the *post hoc* analyses between activated conditions, as unactivated were expected to be different from activated samples. Differences between unactivated conditions (*in vitro*) and between PBS controls (*in vivo*) were reported when significantly different. GraphPad Prism software was used for all statistical analyses. Data are expressed as mean ± standard error of mean (SEM) with statistical significance: ^*^p < 0.05, ^**^p < 0.01, ^***^p < 0.001, ^****^p < 0.0001, NSD, no significant difference.

## Results

### IL-33 Activation Induces Glycolysis and OX PHOS

To determine the effects of IL-33 on mast cell metabolism, we analyzed extracellular acidification rate (ECAR) and proton production rate (PPR) as indicators of H^+^ production and as a surrogate for glycolytic rate. Oxygen consumption rate (OCR) was used as an indicator of mitochondrial OX PHOS. Bone marrow derived mast cells (BMMC) were measured at baseline, following IL-3/SCF addition, and following IL-33 activation. ECAR, PPR, and OCR were all significantly elevated following growth factor addition and IL-33 activation (Figure 1A). We should note that these measures were only significantly elevated with IL-33 activation in the presence of IL-3 and SCF (data not shown). It is known that IL-33 co-stimulation with SCF is necessary for IL-33-induced cytokine production(33), and thus IL-33-mediated changes in glycolysis, like cytokine production, seem to be dependent upon SCF (and possibly IL-3) signaling. We confirmed the induction of glycolysis by IL-33 with measures of glucose uptake and lactate export. Following IL-33 activation for 16 h, glucose uptake and lactate export were calculated using concentrations in the cell supernatants from the IL-33 activated and control groups. IL-33 activation significantly increased glucose uptake and lactate export (Figure 1B), supporting enhanced glycolysis as measured by ECAR and PPR.

Lipopolysaccharide (LPS) is an innate activation signal that shares downstream signaling cascades with IL-33 (34). LPS has been shown to increase macrophage and dendritic cell glycolysis, similar to the IL-33 effects we observed in Figures 1A,B (2, 35, 36). LPS effects occur in 2 stages: a rapidly increased glycolytic enzyme activity and inhibition of OX PHOS, and a prolonged increase in enzyme expression (2, 35, 36). To determine if IL-33 has an effect on glycolytic enzyme expression, BMMC were activated for 8 h ± IL-33. IL-33 induced a modest but consistent increase in hexokinase (HK)2 and pyruvate kinase (PK)M1/2 expression (Figure 1C). These data suggest that IL-33 activation increases both glycolysis and OX PHOS.

### Glycolytic Inhibition Suppresses Cytokine Production Following IL-33 Activation

**Figure 2:**
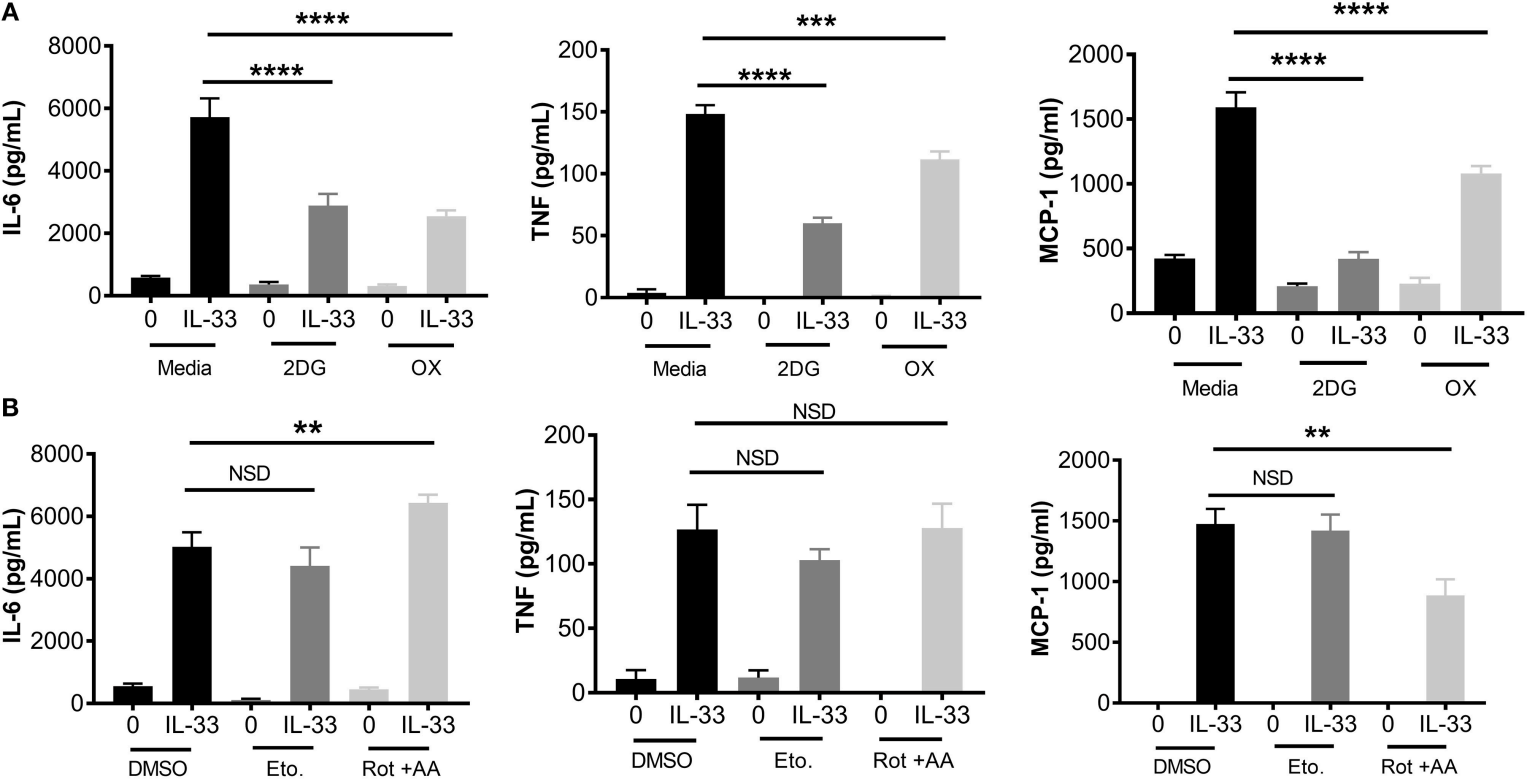
Glycolytic inhibition suppresses cytokine production following IL-33 activation. **(A)** BMMC were treated with 2DG (1 mM) or OX (20 mM) for 1 h prior to IL-33 (50 ng/mL) activation for 16 h. Cytokines were measured in supernatant by ELISA. **(B)** BMMC were treated with DMSO (vehicle control), etomoxir (Eto, 200 μM), or rotenone+antimycin A (Rot+AA, 1 μM) for 1 h prior to IL-33 (50 ng/mL) activation for 16 h. Cytokines were measured in supernatant by ELISA. Data are means ± SEM of 3 populations analyzed in triplicate, representative of 3 independent experiments. ^**^p < 0.01, ^***^p < 0.001, ^****^p < 0.0001, NSD, no significant difference.

To determine the importance of glycolysis for IL-33-mediated mast cell function, we employed chemical antagonists. BMMC were treated ± the glycolytic inhibitors 2DG (1 mM) or OX (20 mM) for 1 h prior to IL-33 activation for 16 h. Importantly, there was no detectable change in cell viability at these doses over the duration of the experiment (data not shown). Both 2DG and OX significantly suppressed IL-33-induced IL-6, TNF, and MCP-1 (Figure 2A). Additionally, we used chemical antagonists to determine the importance of OX PHOS. BMMC treated for 1 h ± etomoxir (Eto, 200 μM), inhibiting fatty acid oxidation, or rotenone and antimycin A (Rot+AA; 1 μM), inhibiting complex I and II of the ETC, had no effect on IL-6 and TNF production (Figure 2B). Rotenone and antimycin A did suppress MCP-1. Rotenone and antimycin A were used at the highest dose at which they did not increase cell death over 24 h, similar to concentrations published to suppress IgE-mediated signaling in mast cells (18). Together with data from Figure 1, these results suggest that the increase in glycolysis following IL-33 activation is functionally important for cytokine production in mast cells and that MCP-1 production may have slightly different signaling and metabolic controls.

### Glycolytic Inhibition Suppresses ERK Phosphorylation, NFκB-Mediated Transcription, and ROS Production

**Figure 3:**
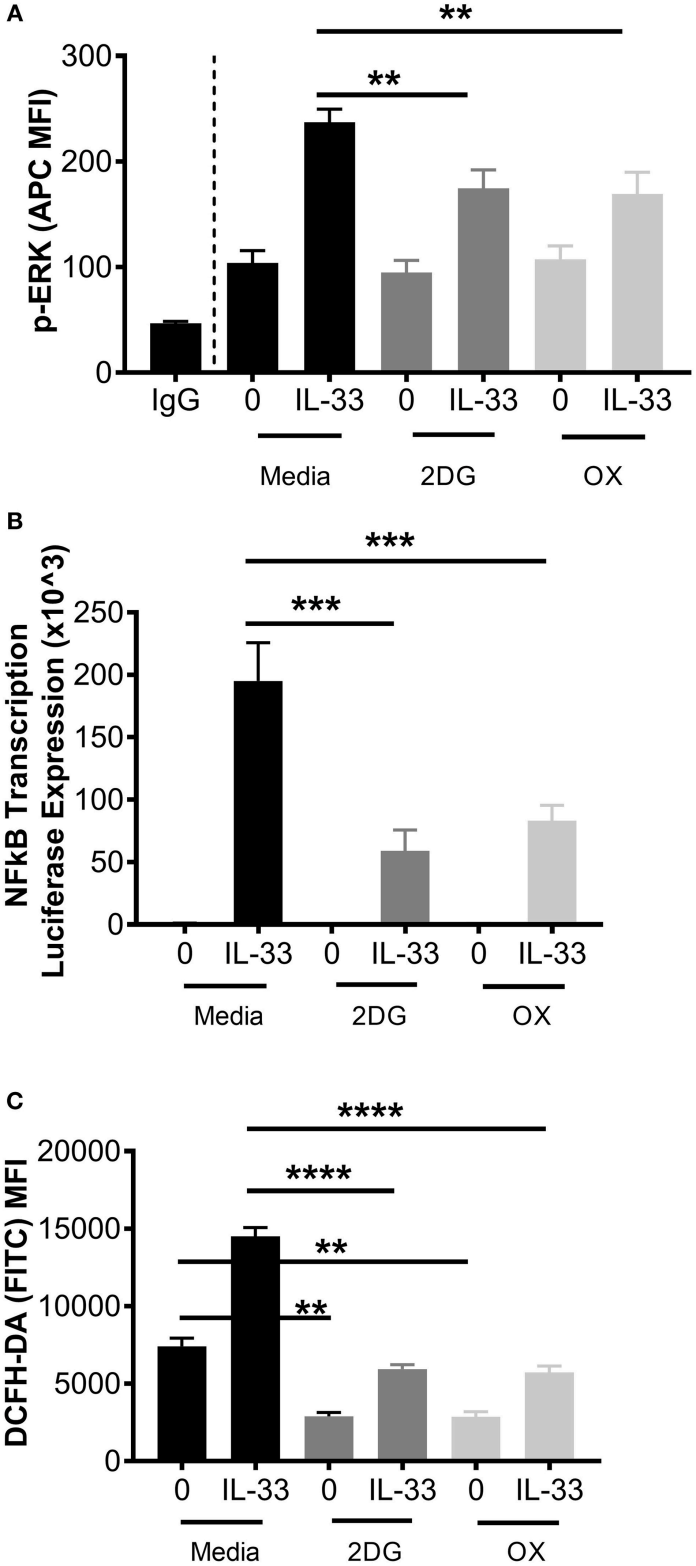
Glycolytic inhibition suppresses ERK phosphorylation, NFκB transcription, and ROS production. **(A)** BMMC were treated with media, 2DG (1 mM) or OX (20 mM) for 1 h and activated +/– IL-33 (100 ng/mL) for 15 min. p-ERK was analyzed by flow cytometry. **(B)** NFκB-luc transgenic BMMC were treated with 2DG (1 mM) or OX (20 mM) for 1 h and activated for 2 h with IL-33 (100 ng/mL). Luciferase activity was measured with the Promega Luciferase Assay Substrate and Glomax Luminometer, then normalized to the unactivated media control. **(C)** BMMC were treated with 2DG (1 mM) or OX (20 mM) for 1 h and activated for 2 h with IL-33 (50 ng/mL). Oxidative stress was analyzed by DCFH-DA fluorescence by flow cytometry. Data are means ± SEM of 3 populations analyzed in duplicate, representative of 3 independent experiments. ^**^p < 0.01, ^***^p < 0.001, ^****^p < 0.0001.

While these data suggest that glycolytic inhibition reduces cytokine release following IL-33, it is unclear if early receptor signaling events are similarly affected. Therefore, ERK phosphorylation (p-ERK) and NFκB transcriptional activity were measured. BMMC were treated ± the glycolytic inhibitors 2DG or OX for 1 h prior to IL-33 activation for 15 min. Phosphorylation events were determined by flow cytometry. As shown in Figure 3A, 2DG or OX treatment significantly suppressed IL-33-mediated ERK activation. Furthermore, NFκB-luc BMMC bearing a luciferase gene driven by two copies of the NFκB regulatory element were also treated ± 2DG or OX for 1 h prior to IL-33 activation for 2 h. Luciferase expression was measured as a surrogate for NFκB-induced transcription. Similar to ERK activation, 2DG and OX significantly suppressed IL-33-mediated luciferase expression (Figure 3B), suggesting that glycolysis is required for IL-33-induced NFκB function.

We have previously published that IL-33 induced-cytokine production is suppressed by antioxidants such as n-acetylcysteine (37), but ROS production following IL-33 activation has not been investigated. Because glycolysis promotes ROS production by the pentose phosphate pathway (2, 38), we examined the effect of glycolytic inhibition on IL-33-induced ROS production. BMMC were treated ± 2DG or OX for 1 h and activated with IL-33 for 2 h. Oxidative stress was measured by DCFH-DA fluorescence by flow cytometry. IL-33 significantly increased DCFH-DA fluorescence, an effect suppressed by 2DG or OX (Figure 3C). Together, these data suggest that glycolysis contributes to IL-33-mediated signaling and ROS production required for optimal mast cell function.

### Increased ATP Availability Restores Cytokine Production Following Glycolytic Inhibition

**Figure 4:**
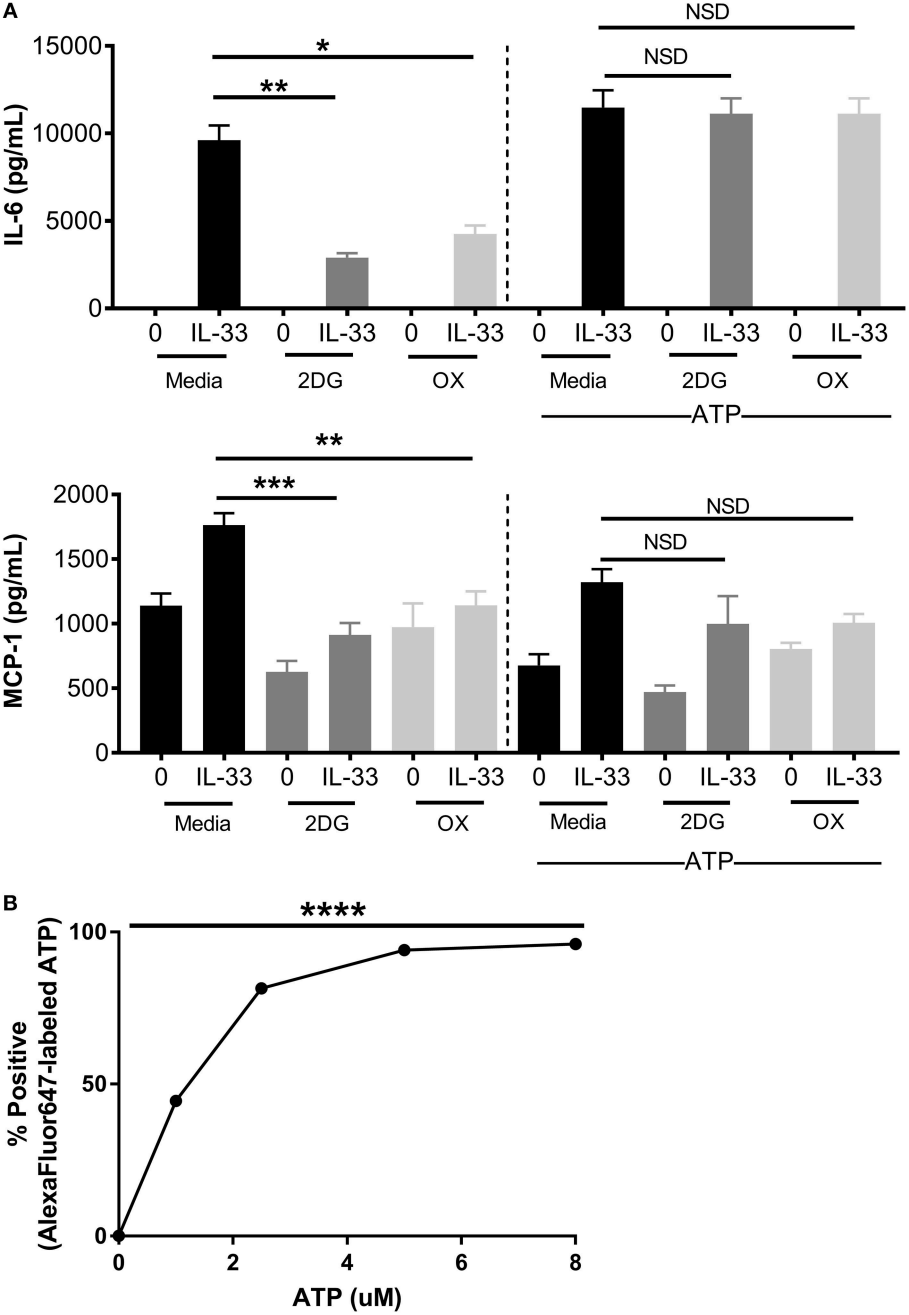
Increased ATP availability restores cytokine production following glycolytic inhibition. **(A)** BMMC were treated with 2DG (1 mM) or OX (20 mM) for 1 h and activated for 16 h with IL-33 (50 ng/mL) and/or ATP (10 μM). Cytokines were measured in cell supernatant by ELISA. **(B)** BMMC were treated with AF647-labeled ATP for 20 min prior to flow cytometric analysis. Data are means ± SEM of 3 populations analyzed in triplicate, representative of 3 independent experiments. ^*^p < 0.05, ^**^p < 0.01, ^***^p < 0.001, ^****^p < 0.0001, NSD, no significant difference.

ATP is required for kinase activity, tRNA synthetase function, ion transport, and chromatin remodeling, all of which play a role in cell signaling and cytokine production. In addition to providing ATP; glycolysis also generates pentose phosophate pathway and Kreb's cycle intermediates used for nucleotide, amino acid, and lipid synthesis (2). To determine if ATP alone is sufficient to restore cytokine production during glycolytic blockade, BMMC were treated for 1 h ± 2DG or OX prior to activation with IL-33 ± ATP (10 mM) for 16 h. While 2DG or OX significantly suppressed IL-6 and MCP production, cytokine production was not significantly suppressed with increased ATP availability (Figure 4A). ATP alone had no effect on cytokine secretion at the concentration used (Figure 4A), and diffused into the cell at low concentrations within 20 min (Figure 4B). Together, these data suggest that ATP availability can maintain cytokine production during glycolytic blockade, supporting the theory that IL-33-induced glycolysis yields ATP that is critical for inflammatory function.

### Glycolytic Inhibitors Suppress IL-33-Induced Neutrophil Recruitment and Cytokine Production *in vivo*

**Figure 5:**
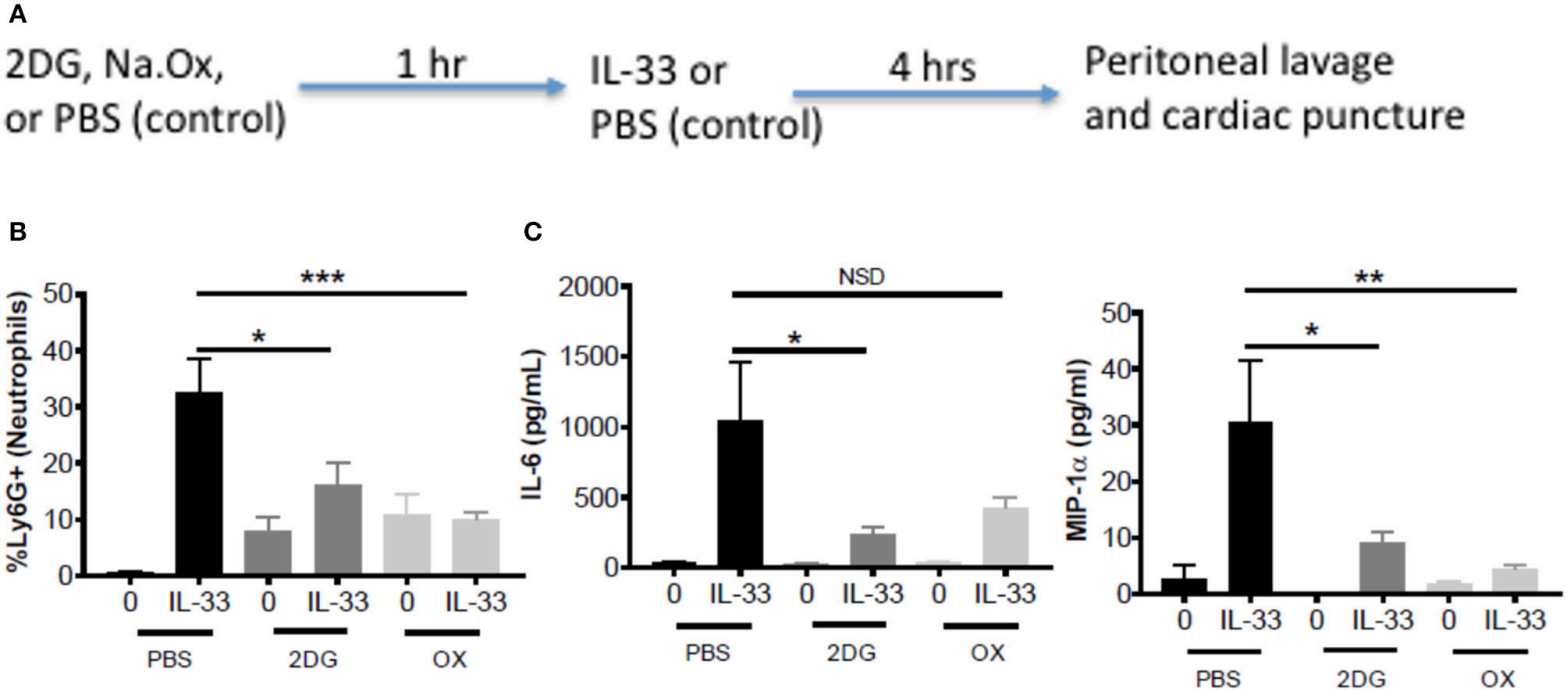
Glycolytic inhibitors suppress IL-33-induced neutrophil recruitment and cytokine production *in vivo***. (A)** Schematic diagram: C57BL/6J mice (age and sex matched males and females) were IP injected with 2DG (750 mg/kg), OX (15 mg/kg), or PBS. After 1 h, mice were injected IP with IL-33 (1 μg) or PBS. All groups were sacrificed after 4 h. **(B)** Peritoneal lavage was used to collect cell infiltrates and analyze neutrophil (Ly6G+) recruitment via flow cytometry. **(C)** Cardiac puncture was used to collect plasma for cytokine analysis via ELISA. Data are means ± SEM of 2 mice/control group and 5 mice/IL-33-peritonitis group analyzed in triplicate (flow cytometry) and duplicate (ELISA), representative of 2 independent experiments. ^*^p < 0.05, ^**^p < 0.01, ^***^p < 0.001, NSD, no significant difference.

To test the importance of IL-33-induced glycolysis *in vivo*, we used a model of IL-33-induced peritonitis in which neutrophil recruitment is mast cell-dependent (39). As shown in the schematic (Figure 5A), mice were IP injected with 2DG (750 mg/kg), OX (15 mg/kg), or PBS (control). After 1 h, mice were injected IP with IL-33 (1 μg) or PBS. After 4 h, peritoneal lavage and cardiac puncture were harvested. 2DG and OX significantly suppressed IL-33 induced neutrophil (Ly6G^hi^) recruitment into the peritoneum compared with the PBS control group (Figure 5B). Similarly, 2DG and OX reduced plasma IL-6 and MIP-1α (Figure 5C). We should note that 2DG and OX induced neutrophil recruitment independent of IL-33 (Figures 5B,C). These data suggest the suppressive effects of glycolytic inhibition extend to IL-33 activation *in vivo*, but also point to limitations regarding their use.

### IL-33 and AMPK-SIRT1 Are Antagonistic Pathways

While 2DG has been used in a Phase I trial for cancer treatment, there have been off-target effects reported. Additionally, the C_MAX_ reported for the recommended Phase II 2DG dose was 450 mM (for 45 mg/kg) (40), well below the doses used in our model. Fatigue, dizziness, and dose-dependent cardiac QTc prolongation were also observed in a few patients (40). Similarly, OX is known to have poor cell membrane permeability and concentrations sufficient for LDH suppression cannot be reached *in vivo* (41). Due to the limitations of these inhibitors for future clinical use, we became interested in AMPK as another way to target glycolysis in IL-33-related diseases.

**Figure 6:**
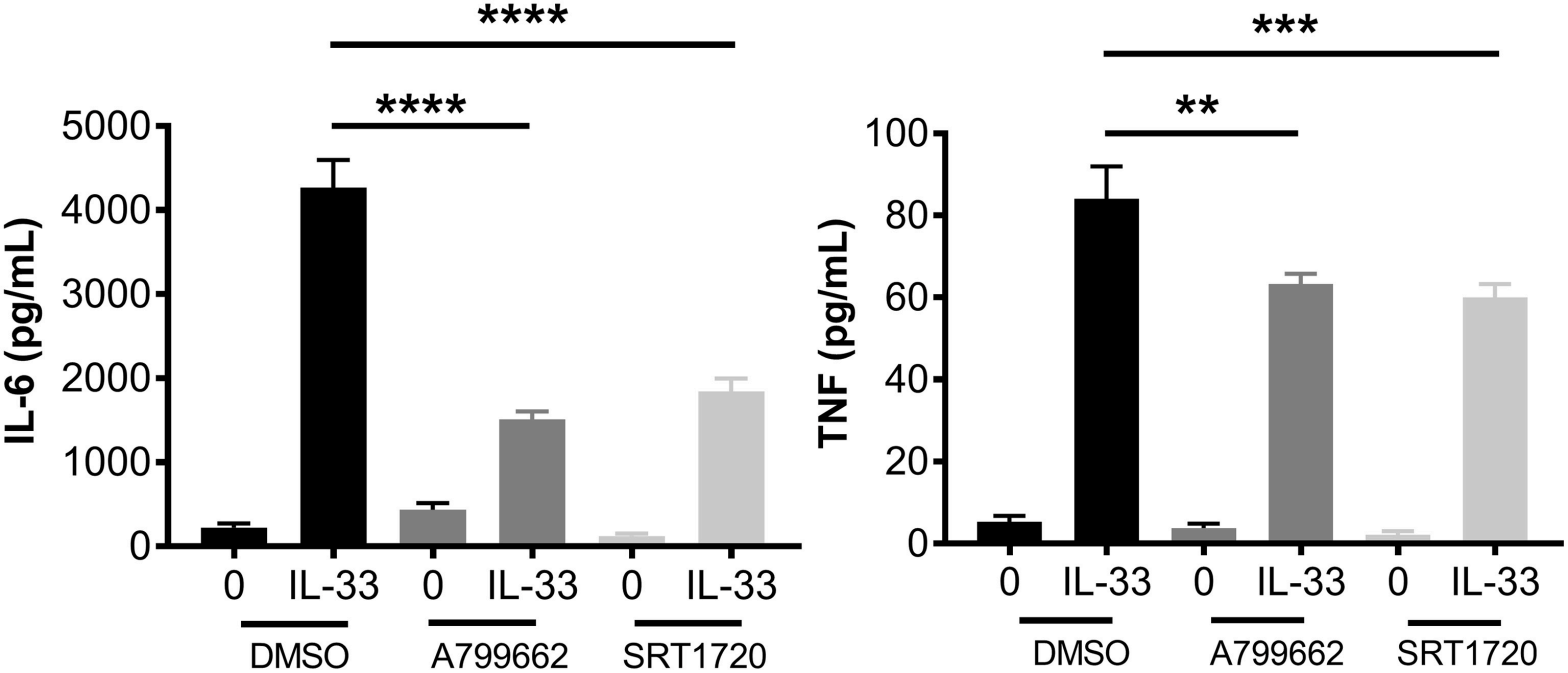
IL-33 and AMPK-SIRT1 are antagonistic pathways. BMMC were treated with DMSO (vehicle control), A799662 (AMPK agonist, 100 μM) or SRT1720 (SIRT1 agonist, 5 μM) for 1 h and activated with IL-33 (50 ng/mL) for 16 h. Cytokines were measured in supernatant by ELISA. Data are means ± SEM of 3 populations analyzed in triplicate, representative of 3 independent experiments. ^**^p < 0.01, ^***^p < 0.001, ^****^p < 0.0001.

AMPK is known for its role in energy sensing, activated in response to fasting and exercise. AMPK switches the cell from anabolic pathways to catabolic pathways, utilizing all potential energy in the form of glucose and lipids by both glycolysis and OX PHOS in liver, kidney, and skeletal muscle (2). Interestingly, AMPK increases OX PHOS but inhibits glycolysis in immune cells, perhaps because of the anabolic effects of glycolysis (2, 42). To determine the effects of AMPK on IL-33-induced cytokine production, BMMC were treated with A799662 (AMPK agonist; 100 μM) or SRT1720 (an agonist of the APMK target SIRT1; 5 μM) for 1 h prior to activation with IL-33 for 16 h. Both agonists significantly reduced IL-33-mediated IL-6 and TNF production (Figure 6). These data suggest that activating AMPK or its downstream signaling pathways can suppress IL-33-induced mast cell activation, mirroring the effects of glycolytic inhibition.

### Metformin Suppresses IL-33-Induced BMMC Activation as Well as Neutrophil Recruitment and Cytokine Production *in vivo*

**Figure 7:**
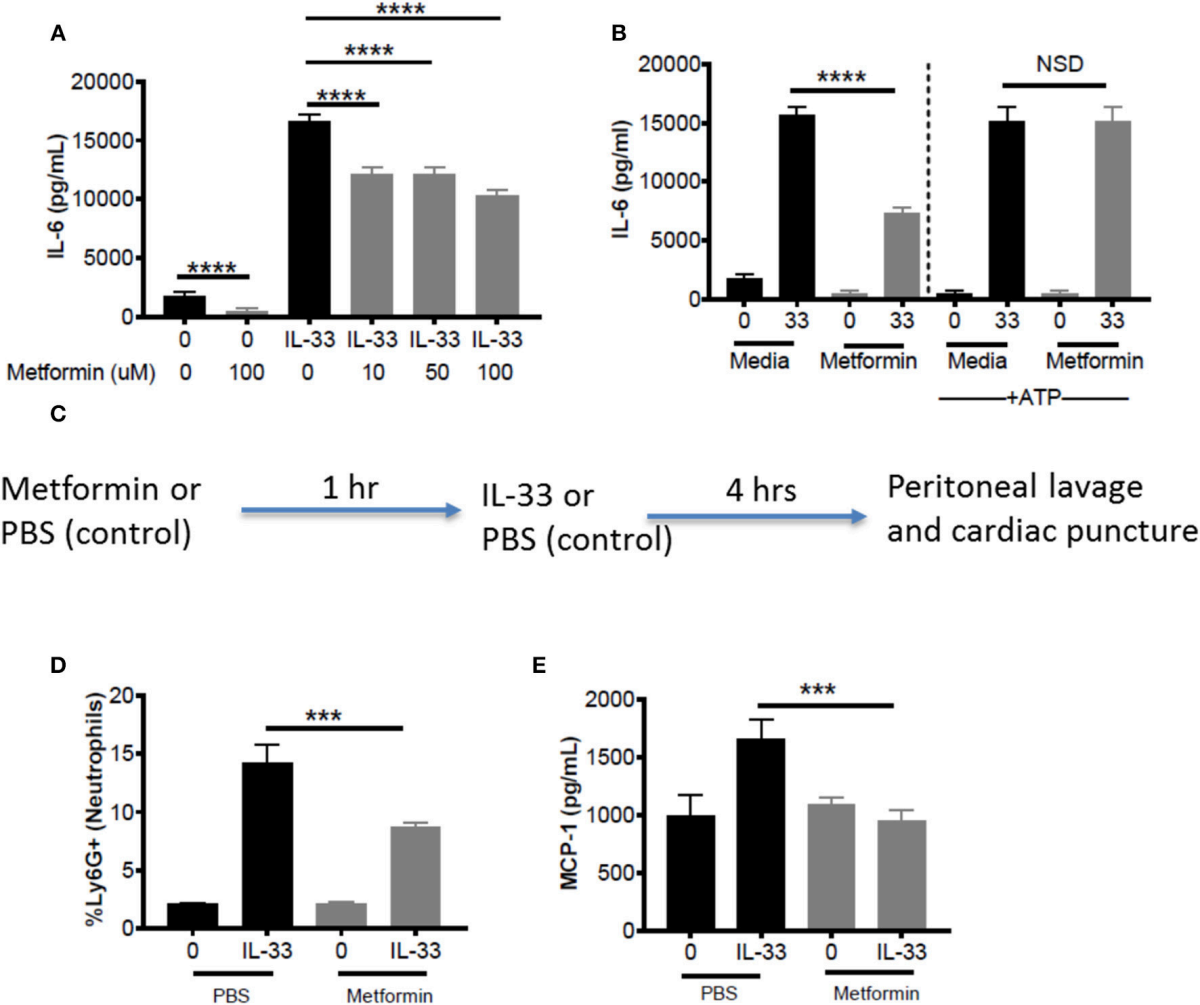
Metformin suppress IL-33-induced BMMC activation as well as neutrophil recruitment and cytokine production *in vivo*. **(A)** BMMC were treated ± metformin (10, 50, 100 μM) for 24 h and activated with IL-33 (50 ng/mL) for 16 h. **(B)** BMMC were treated ± metformin (Met, 100 μM) for 1 h and activated with IL-33 (50 ng/mL) ± ATP (10 μM) for 16 h. Cytokines were measured in the supernatant by ELISA. Above data are means ± SEM of 3 populations run in triplicate, representative of 3 independent experiments. **(C)** Schematic diagram: C57BL/6 males and females were injected intraperitoneal (IP) with metformin (100 mg/kg) or PBS. After 1 h, mice were injected IP with IL-33 (1 μg) or PBS. All groups were sacrificed after 4 h. **(D)** Peritoneal lavage was used to collect cell infiltrates and analyze neutrophil (Ly6G+) recruitment via flow cytometry. **(E)** Cardiac puncture was used to collect plasma for cytokine analysis via ELISA. *In vivo* data are means ± SEM of 3 mice/control group and 8 mice/ peritonitis group analyzed in triplicate (flow cytometry) or duplicate (ELISA), representative of 2 independent experiments. ^***^p < 0.001, ^****^p < 0.0001.

Metformin is an FDA-approved AMPK activator, widely prescribed for the treatment of diabetes. This offers a clinically-relevant means of assessing how AMPK activation affects IL-33 function. First, BMMC were treated with metformin at physiological doses (10, 50, 100 μM) (43, 44) for 24 h before IL-33 stimulation for 16 h. For all metformin doses, we observed significant suppressive effects (Figure 7A). Similar suppression was observed with 1-h treatment, albeit at higher doses (data not shown). To confirm that the effects of metformin were due to reduced glycolysis and ATP availability, BMMC treated with metformin (100 mM) for 24 h were activated with IL-33 ± ATP (10 mM) for 16 h. ATP reversed the suppression by metformin (Figure 7B). These results suggest that suppressing glycolysis and ATP production by increasing AMPK activity with metformin is an effective way to limit cytokine production following IL-33 activation.

To establish proof of principle *in vivo*, metformin treatment was used in the IL-33-induced peritonitis model. As shown in the schematic (Figure 7C), mice received metformin (IP, 100mg/kg, ~100 μl) or PBS (control, 100 μl). After 1 h, both groups of mice were injected IP with IL-33 (1 μg, 100 μl) or PBS (100 μl). Peritoneal lavage and cardiac puncture were harvested after 4 h. Metformin significantly suppressed IL-33-mediated neutrophil (Ly6G^hi^) recruitment into the peritoneum compared with the PBS control group (Figure 7D). Furthermore, metformin significantly reduced plasma MCP-1 (Figure 7E). These data support the theory that indirectly targeting glycolysis with an AMPK agonist can suppress IL-33-mediated inflammation *in vivo*.

## Discussion

Immune cell metabolism is closely linked to phenotype and effector functions. While T cell and macrophage metabolism have been highly studied over the past decade, mast cell metabolism and IL-33-mediated activation have received little attention. This study is the first to report that IL-33 increases glycolysis, generating ATP that is required for subsequent inflammatory cytokine production. Targeting glycolytic ATP production by inhibiting glycolysis with 2-DG and OX, or by activating AMPK with metformin was sufficient to reduce IL-33-mediated effects *in vitro* and *in vivo*. With increased ATP availability, these inhibitors had little effect on cytokine production, highlighting the importance of this glycolytic product. These data advance our understanding of IL-33 function and suggest that regulators of glycolysis, like AMPK, may be potential targets for treating inflammatory diseases involving IL-33.

Previous to this report, only one paper using small-cell lung cancer (SCLC) cells reported metabolic effects of IL-33, showing increased glucose uptake and lactate export (45). This work is the first to note glycolytic requirements for IL-33 activation in immune cells. Additionally, we observed a slight increase in OX PHOS. We hypothesize that IL-33 signaling in other ST2^+^ cells, including Th2 and ILC2 cells, would also require glycolysis for optimal function, but this remains to be determined. Using the Seahorse analyzer, we observed increased glycolysis immediately upon IL-33 stimulation, likely due to increased enzyme activity. Additionally, metabolic enzyme expression was moderately elevated at 8 h, and glucose uptake and lactate export remained different at 16 h. While we noted an increase in HK2 and PKM1/2 expression, Wang et al. observed increased Glut1 surface expression in SCLC cells (45), suggesting that IL-33 may regulate the expression of many different proteins important for glycolysis.

Our data suggest a functional role for enhanced glycolysis following IL-33 activation. In BMMC, the glycolytic inhibitors 2DG and OX suppressed IL-33-mediated ERK activation, NFκB transcription, ROS production, and cytokine secretion. These data are consistent with other findings, as 2DG and OX inhibit IgE receptor-induced cytokine secretion in rat mast cells (16), cytokine mRNA and protein secretion, cytolytic activity, and cell cycle progression in CD8 T cells (46), and ERK phosphorylation in SCLC cells (47). It is important to note that inhibiting glycolysis also reduces glucose consumption and pyruvate availability for Kreb's cycle and the ETC, which is likely why others have noted OX PHOS suppression with 2DG (48). This effect may be greater with 2DG, as OX may push some pyruvate into the mitochondria for utilization, and should be accounted for with future studies. Thus, determining the importance of glycolysis vs. OX PHOS when analyzing inhibitor studies has challenges. Our interpretations of the data are that glycolytic ATP product is paramount for IL-33-induced mast cells responses. This conclusion is based on (1) ATP-mediated reversal of glycolytic suppression and (2) the consistent inhibitory effects of glycolytic inhibitors in contrast to inconsistent or lack of effects when blocking OX PHOS and ETC function. Because 2DG and OX suppress IL-33-mediated cytokine production and neutrophil recruitment *in vivo*, our data suggest that glycolytic ATP plays a critical role in IL-33-mediated inflammation.

ATP, the primary product of glycolysis, provides a phosphate group and energy for kinases (45, 49) and enzymes involved in signaling and transcription. Our data show that exogenous ATP can restore IL-33-induced cytokine production in the presence of glycolytic inhibitors. It is important to note that the effects on IL-6 appear more dramatic than MCP-1. Along with our data suggesting that OX PHOS blockade suppresses MCP-1, MCP-1 production likely utilizes different signaling cascades and transcription factors and may therefore require more ATP for transcription or additional products from glycolysis and subsequent pathways. Nevertheless, we see that ATP reverses OX suppression from 35 to 23%, suggesting at least partial reversal. We hypothesize that glycolytic blockade reduces ATP availability and thus, ATP available for kinase phosphorylation and signaling through NFκB. Reduced signaling then leads to reduced cytokine production, suggesting that glycolytic blockade effects many downstream signaling events. We note here that low dose ATP was shown to diffuse into the cell with no concomitant IL-6 or MCP-1 production, suggesting this effect is independent of P2X receptors, which are activated at concentrations 10-fold higher (data not shown). Together with the above data, these results support the hypothesis that mast cells require glycolytic ATP production for IL-33-induced function, and suggest that glycolytic blockade can suppress IL-33-mediated inflammation *in vivo*.

Interestingly, we show here that IL-33 increases glycolysis in mast cells, increasing both lactate export and H^+^ ion production, and we have previously published that lactic acid suppresses IL-33-mediated cytokine production in mast cells (31). From this we hypothesize that lactic acid increases with IL-33 activation and may act as a feedback regulator of IL-33 activation. There is evidence that glycolysis is elevated in both adaptive and innate immune cells with pro-inflammatory stimulation (1, 6), that lactic acid increases in inflammatory environments (50–54), and that lactic acid suppresses pro-inflammatory immune functions and shifts macrophages to a wound healing phenotype (55). Together, these results suggest that the feedback inhibition may play a larger role in inflammation, cancer, sepsis, asthma, and wound healing. Understanding these pathways and inherent feedback mechanisms may help us to better develop and dose drug treatments for use in different inflammatory diseases.

While 2DG and OX have been used in humans, there is little potential for their clinical use due to dose and side effect limitations. Our data suggest that activating AMPK, a mediator of cell metabolism, may be effective in IL-33-related diseases. There has been no evidence to directly link AMPK to IL-33 in immune cells, although systemic administration of an anti-ST2 antibody increased AMPK phosphorylation in the renal parenchyma of mice (56). We show that activating AMPK with metformin or the specific agonist A799662 suppressed IL-33-induced cytokine production. Furthermore, activating SIRT1, a deacetylase downstream of AMPK known to play a suppressive role in signal transduction and glycolysis (57–59), similarly suppressed cytokine production. This provides another possible clinical target for modulating IL-33-induced inflammatory responses and suggests that SIRT family members should be studied as a target in IL-33-related disease. ATP reversed metformin effects, supporting the theory that inhibiting glycolytic ATP production is the primary mechanism of action. With metformin use *in vivo*, we also provide proof of principle for its utility in IL-33-related pathologies. These data support the idea that metformin provides anti-inflammatory effects beyond lowering blood glucose, and suggest that AMPK is a rational target for suppressing IL-33-mediated cytokine production and effector functions *in vivo*.

Interestingly, IL-33 increased both glycolysis and OX PHOS in mast cells, similar to IgE signaling. However, blocking OX PHOS did not influence IL-6 or TNF production, in contrast to data reported with IgE-mediated activation (18). Lawrence Kane's group observed that rotenone (blocking complex I) suppressed IL-6 production and degranulation in IgE-mediated activation (18), yet in our studies with IL-33, rotenone and antimycin A (blocking both complex I and complex III) had no effect on IL-6 or TNF secretion. Interestingly, we do find effects on MCP-1. The dose of rotenone (1 mM) was the same in each study. This suggests important differences regarding the use and control of energy pathways in the IgE and IL-33 signaling cascades and potential differences in the signaling, transcription, and translation required for the production of different cytokines.

Future work should examine the signaling mechanisms directly responsible for changes in metabolism and cytokine transcription. Transcription of both glycolytic enzymes and cytokines are linked to HIF-1α following LPS activation. TLR4 signaling can induce NADPH oxidase activity and ROS production, which stabilizes HIF-1α (60). ASK1, p38, and ERK signaling can also contribute to HIF-1α accumulation (61–63), which suggests that similar pathways may play a role in IL-33-mediated changes. Furthermore, glycolysis induction is known to provide intermediates for lipid synthesis and histone acetylation (2, 6), in addition to generating ATP and ROS. The importance of these intermediates should be examined in the context of IL-33 activation.

This work emphasizes the importance of glucose metabolism in mast cell function, supporting recent publications by Ehud Razin's and Lawrence Kane's labs, which examined IgE activation of mast cells (18, 19). IL-33 activation increases glycolysis to provide ATP and ROS for optimal receptor signaling, cytokine production, and effector functions. Direct glycolytic blockade and metformin-induced AMPK activation were sufficient to reduce cytokine production both *in vitro* and *in vivo*. Together, these data advance our understanding of IL-33 activation and suggest that AMPK and glycolysis are potential targets for treating IL-33-mediated disease.

## Author Contributions

All authors assisted in experimental design. HC, MT, TH, NP, EB, and BB conducted experiments and performed initial analyses. HC and JR did detailed analyses and created most data figures. HC and JR wrote the manuscript with editing assistance from all co-authors.

### Conflict of Interest Statement

The authors declare that the research was conducted in the absence of any commercial or financial relationships that could be construed as a potential conflict of interest.
